# A study protocol for rural tribal school partnerships for the primary prevention of obesity among American Indian youth (P^2^)

**DOI:** 10.3389/fpubh.2026.1957377

**Published:** 2026-10-26

**Authors:** Regina Eddie, Velia Leybas Nuño, Brenda Charley, Betsy C. Wertheim, Nick Calles, Brighten Crawford-Martin, Angela Yung, Raeshaun Greenwood, Seth Dominguez

**Affiliations:** 1 Sanghi College of Nursing, Northern Arizona University, Flagstaff, AZ, United States; 2 University of Arizona Cancer Center, Tucson, AZ, United States; 3 Department of Health Promotion Sciences, Mel and Enid Zuckerman College of Public Health, Tucson, AZ, United States; 4 Department of Health Sciences, Northern Arizona University, Health Sciences, Flagstaff, AZ, United States

**Keywords:** American Indian/Alaska native, cancer prevention, nutrition, obesity prevention, physical activity, policy, school

## Abstract

Obesity among American Indian youth remains a critical health disparity, placing them at elevated risk for chronic diseases such as diabetes, cardiovascular disease, and obesity-related cancers. School environments in Tribal communities are uniquely positioned to support obesity prevention through structured health education, physical activity opportunities, and school meal programs and school wellness policy initiatives. Yet, school-based obesity prevention research in American Indian communities remains limited, and have not been as successful in improving weight status in AIAN youth, particularly in leveraging the requirement of school wellness policies as participants in the National School Lunch Program. We describe the procedure and rationale for evaluating the Rural Tribal School Partnership for the Primary Prevention of Obesity (P2) project, a multi-component health promotion curriculum intervention addressing nutrition and physical activity through three integrated components – classroom, food services and policy. We will evaluate the efficacy of P2 for improving BMI, nutrition and physical activity knowledge and behaviors (primary outcomes) among American Indian 4th grade students in rural, Tribal communities. We will employ a cluster, non-randomized design across four schools, with one intervention arm and one control arm. Enrolled 4th grade youth will complete pre- (start of school year) and post-intervention (end of school year) measurements. Secondary outcomes include physical activity tracking, dietary recall, student perception of school environment policies and practices, objective fruit and vegetable intake using Veggie Meter^®^. Study findings will inform best practices and a scalable culturally responsive health promotion curriculum model for rural and American Indian serving schools.

**Clinical trials registration:**

NCT06864468, https://register.clinicaltrials.gov/prs/beta/records.

## 1 Introduction

Youth in the United States comprise a dynamic and diverse population. American Indian/Alaska Native youth represent a unique population affected by a long history of cultural, social, and environmental disruption and change (1, 2). This history has contributed to some of the most significant health disparities seen in any youth population today, including high and persistent rates of childhood overweight and obesity (3). For instance, Bullock and colleagues report an estimated 30% of American Indian/Alaska Native youth (age 2–19 years) experience obesity, nearly double their U. S. peers of 17% (3). The onset of overweight and obesity can have short and long lasting implications on health, such as increasing the risk for Type 2 diabetes, heart disease, and cancer (4).

One approach to improving these health challenges and reaching American Indian/Alaska Native youth is to partner with schools. Although schools are primarily designed to directly develop children’s academic knowledge and skills, their role extends well beyond academics. Children spend 6–8 h per day at school for most of the week, making schools critical settings for engaging American Indian/Alaska Native youth in healthy behaviors that can impact health outcomes (5). The rural nature of many Tribal communities adds barriers to promoting healthy behaviors among American Indian/Alaska Native youth. These barriers include limited infrastructure, economic hardship, geographic isolation, and reduced access to healthy foods due to food desert conditions (6, 7).

In these contexts, schools may be the only setting where children can access nutritious meals and opportunities for physical activity. Current physical activity guidelines recommend children and adolescents (age 6–17 years) should achieve at least 60 min of moderate to vigorous physical activity daily (8); however, limited data suggest that many American Indian youth are not meeting this standard (9–11).

Dietary patterns among American Indian youth are similarly concerning. A 2022 report found that American Indian/Alaska Native youth consumed 15% less fruit and 30% less vegetables per day compared to other youth (12). Further, 63.1% of American Indian/Alaska Native youth drank sugar-sweetened beverages at least once a week compared to 57.7% of the total population (12).

### 1.1 School wellness policies

School wellness policies (SWPs) emerged as an endeavor to improve school nutrition and physical activity environments and to promote child health and wellbeing. The Child Nutrition and WIC Reauthorization Act of 2004 was the first national policy to require schools and school districts receiving federal funding under the National School Lunch Program to develop and implement an SWP (13, 14). In 2010, the passage of the Healthy, Hunger-Free Kids Act (HHFKA) set forth new SWP requirements aimed at strengthening the nutrition quality of breakfasts and lunches, as well as other foods and beverages sold or served through á la carte options, classroom parties, fundraisers, and school events. Notably, the HHFKA also introduced transparency requirements governing the development, implementation, evaluation, and monitoring of SWPs (14).

Many schools in Tribal communities participate in the National School Lunch Program under the Community Eligibility Provision (CEP), a funding option for high poverty schools that provides free breakfast and lunch to all students (15). These schools are also required to adopt and implement a SWP, which can be a powerful mechanism for holding schools accountable for supporting and encouraging healthy behaviors, as they set clear expectations for nutrition education, foods served and sold, physical activity, and physical education practices (Table 1) (5).

**Table 1:** Minimum standards included in a school wellness policy.

Specific goals	Nutrition promotion and education, physical activity, and other school-based activities meant to promote student wellness
Standards and nutrition guidelines for all foods and beverages	All consumables sold to students on school campuses must be consistent with federal regulations for:
	School meal nutrition standards Smart Snacks in School nutrition standards
Standards for all foods and beverages provided, but not sold, to students	Consumables made available to students through classroom parties, snacks brought by parents, or other foods provided as incentives
Policies for food and beverage marketing	Advertising consumables that meet the Smart Snacks in Schools nutrition standards
Description of public involvement, public updates, policy leadership, and evaluation plan	

Data adapted from Food and Nutrition Service (2025, September 9). Local school wellness policies. U. S. Department of Agriculture. https://www.fns.usda.gov/tn/wellness-policy

Interestingly, systematic reviews concluded that there is no single approach to addressing the complexity of childhood obesity but rather combining intervention components has better outcomes. For instance, Hassan et al. (16) found that physical activity alone was more effective than diet and nutrition alone in improving BMI. Further, combining physical activity with dietary guidance was more effective than either strategy alone, and multiple component interventions were the most effective in reducing BMI z-scores (16). Similarly, Klein (17) analyzed 27 studies, concluding that both physical activity and nutritional changes offer meaningful benefits. Specifically, nutritional change through education alone or education paired with cooking lessons was the most effective standalone strategy, followed by physical activity exceeding 40 min sustained over 9 months. Pairing physical activity with nutrition change offered the highest likelihood of improving obesity measures.

Comparable results have not been documented in American Indian communities, as research specifically targeting American Indian youth in school settings remains limited, and school-based programming and interventions have not been as successful in improving weight status in American Indian/Alaska Native youth compared to other groups. Pathways—the first large-scale, culturally grounded, randomized school-based intervention across seven Tribal communities—is considered a landmark study in this area (18). Its multi-component program integrating classroom, family, school food services, and physical education components laid the foundation for how school-based obesity research in American Indian/Alaska Native communities can be conceptualized and designed. Though it did not achieve statistically significant reductions in percent body fat, secondary successes in diet quality, knowledge, and physical activity were documented. Beyond Pathways, the evidence is sparse. Wann and colleagues (19) identified only two school-based studies among six obesity prevention studies in American Indian/Alaska Native youth, and none reported improvements in BMI. Similarly, Fleischhaker et al. (20) found no evidence of weight-related changes across 20 physical activity intervention studies; among the three school-based studies, only one reported making school policy changes, but with no detail provided. Andreo and Andrade (21) evaluated 12 nutrition-related interventions targeting American Indian/Alaska Native youth, with only three involving school food environments; while slight improvements in nutrition knowledge, dietary adherence, and reduction in dietary fat in school meals were noted, none produced changes in child weight outcomes.

Taken together, several important gaps emerge. Many of the studies, including Pathways, date to the early and mid 2000s, and none of the studies examined multilevel interventions designed to change school policies. Further, to our knowledge, since the HHFKA of 2010, Eddie et al. (22) is the only group to have examined and documented school food environment policies and practices in schools on a Tribal reservation. That study revealed significant gaps in school food environments, including a lack of active school wellness councils, limited principal awareness of nutrition standards, and few formal policies around fundraising and classroom parties, underscoring the urgent need for stronger school-level policies and practices (22).

To address these gaps, the goal of our study is to partner with five separate schools on the Navajo Nation to design and implement a culturally tailored, multi-component intervention within the current policy context of the HHFKA of 2010. A Two-Eyed Seeing framework will be applied as a guiding principle to integrate Indigenous and Western ways of knowing throughout the study (23). This paper describes the protocol for three aims: (1) to conduct a formative assessment to evaluate the capacity and resources needed to implement a train-the-trainer model for fourth grade and physical education teachers, as well as food service personnel; (2) to assess changes in BMI, nutrition and physical activity knowledge, and behaviors among fourth grade students; and (3) to assess changes in school wellness policies from pre to post-intervention using the Wellness School Assessment Tool (WellSAT).

## 2 Study methods and analysis

### 2.1 Study setting

In 2023, there were 559,819 American Indian/Alaska Native children living in the United States (24). Nearly half of American Indian/Alaska Native people live in seven states, including Arizona (24). Arizona is home to 22 federally recognized tribes, with American Indians comprising 5.1% of the state’s population (25). Located within Arizona is the largest Tribal reservation in the United States – the Navajo Nation – occupied by a people who refer to themselves as the Diné. Spanning across 27,000 square miles in northeast Arizona, northwest New Mexico, and southeast Utah, the Navajo Nation is demographically substantial (26). In 2021, Navajo Nation’s total enrolled population reached nearly 400,000, making it the most populous federally recognized tribe (27). K-12 education on the Navajo Nation is administered through multiple systems including state-run public schools, Bureau of Indian Education schools, tribally controlled contract and grant schools, and charter schools. During the 2025–2026 school year, total K-12 student enrollment was 26,271 across the various school systems (28).

We will recruit five schools located in separate small, rural communities on the Navajo Nation: three small tribally controlled grant schools with total student enrollment that ranges from 100–220 students each, and two large public schools with ~400 elementary students each. The population size of these communities ranges from 300 to 5,000 people (29). The distance between the communities ranges from 45 to 250 miles, with the closest community to research staff being approximately 90 miles.

### 2.2 Study design

We will employ a cluster, non-randomized design across five schools. The five schools were selected based on their interest in and availability to participate as well as the ability to obtain the necessary approvals within the timeframe of data collection at the beginning of the school year. The study includes three small schools, each with one fourth grade classroom and two large schools with multiple fourth-grade classrooms. In the first year of data collection, two schools will serve as intervention sites—one Tribally controlled grant school and one public school—and two other Tribally controlled grant schools will serve as control sites. In the following year, we will recruit another large school as a control site to match the large intervention school, and the two small schools who were controls in year 1 will receive the intervention in year 2. We expect at least 80 and 40 fourth graders in the intervention and control groups, respectively, to complete both pre and post-intervention measurements. Considering a range of ICC values (0.01–0.05), this sample size will allow detection of a 0.64–0.87 standardized difference (difference in the means divided by the standard deviation) between the intervention and control youth in the BMI z-score change (primary endpoint), assuming 80% power and an alpha level of 0.05. We hypothesize that participation in our health promotion interventions will strengthen school-specific wellness policies, and that fourth grade youth in the intervention schools will demonstrate improved health outcomes related to BMI, fruit and vegetable intake, and physical activity.

### 2.3 Study eligibility

Participating schools will include public schools and tribally controlled grant or contract schools on the Navajo Nation that are participants of the National School Lunch Program. Adults taking part in focus groups and key informant interviews must hold relevant participating school positions such as principal/assistant principal, fourth-grade teacher, physical education teacher, health technician, and food service personnel. Further, all adult participants need to speak English, and the need for interpreters or translations of the Navajo language is not anticipated. Adults must complete a consent form. Eligible students must be enrolled in the fourth grade, ages 8–10 years, and plan to attend both baseline and post-intervention assessments. Parent or caregiver consent and student assent will be required for students to participate. We will collect parent consent during recruitment at the Healthy School Carnival. Students may also give consent forms to their fourth grade teacher, who will then provide to study personnel. Assents will also collected at the time of recruitment or on the day of data collection. Signed consents and assents are stored and transported in a study sealed travel case accessible to only study personnel, then returned to locked file cabinets in the principal investigator’s office.

### 2.4 Ethics approvals

All study procedures were approved by Northern Arizona University and Navajo Nation Human Research Review Board (NNHRRB), local school board and community approvals. For one school, agency approval was also secured. A ceded review approval by the University of Arizona Institutional Review Board was also secured. Data collected belongs to NNHRRB and any dissemination requires prior review and approval. To ensure data security, data collected will be stored electronically in the Research Electronic Data Capture (REDCap) platform and password protected. All paper-based study materials will be filed using de-identified code IDs and secured in locked file cabinets within one of the principal investigator’s office, accessible to only authorized study personnel.

#### 2.4.1 Study team and approach

Our current study team represents varied backgrounds including American Indian, Mexican American, and white descent. The study team will employ the guiding principle of Two-Eyed Seeing, an approach that acknowledges both Indigenous and Western ways of knowing and learning to see value in each (23, 30). This bridging of worldviews is not unfamiliar to the Diné people, who, as Chief Manuelito urged in the 1800s, understood that both worldviews held value and that new opportunities could emerge by honoring both ways of knowing.

Further grounding this approach is K’é, a Diné concept based on reciprocity and how one relates to other Diné, to all beings on earth, and how they in turn relate to us. Diné traditions and ways of knowing are built on the understanding and lived application of K’é. This Indigenous-led project will employ k’é at all levels of project management, cultivating reciprocal relationships with research staff, teachers, school staff and administration, and community partners. For example, applying k’é may look like beginning every in-person meeting with Diné introductions – a cultural practice that establishes kinship relationships, which creates a foundation for greetings in the future, and builds trust and respect essential for collaborative research.

### 2.5 Intervention methods

#### 2.5.1 Intervention development and curricula

To launch the project, the study team will conduct semi-structured interviews and focus groups with school personnel at the two intervention schools from early March through the end of May of the first funding year. Participants (n = 10) will include principals, food service personnel, physical education instructors, fourth-grade teachers, health aides, and designated school wellness coordinators. The formative research seeks to accomplish four goals: (a) map each school’s current needs, resources, and capacity to establish a clear picture of the existing landscape; (b) assess feasibility and collaborate with school partners to plan intervention curricula implementation; (c) examine the current food services program and its capacity to prepare and serve healthy foods; and (d) explore what and how students are currently taught regarding health promotion and physical activity, including cultural teachings and practices about health such as serving traditional foods. Formative research is one way we will implement the Two-Eyed Seeing approach. Findings will instruct our intervention components that are designed primarily to address increased fruit and vegetable intake and increased physical activity through three integrated intervention components: (a) classroom curriculum, (b) Diné (Navajo) chef consultation, and (c) wellness policy assessment.

#### 2.5.2 Classroom curriculum

The Primary Prevention of Obesity (P^2^) health promotion curriculum will adapt materials from previous curricula developed for American Indian youth. These include Pathways (18) and The Child Health Initiative for Lifelong Eating and Exercise (CHILE) (31), and structured education sessions from the American Indian Youth Wellness Camp (32). The P^2^ curriculum will consist of one approximately 45–60 min lesson per week for 10 weeks delivered by teachers using the train-the-trainer model. Lessons will target the following objectives: (a) increase knowledge of fruit, vegetable, and physical activity guidelines; (b) increase classroom-based physical activity time; (c) build skills in limiting screen time, deep breathing and relaxation, and reading food labels; (d) connect healthy behaviors to traditional teachings and practices. All intervention schools will participate in the same core lessons. Each lesson will follow a standardized format and include the same components for congruity: title, time length, objectives (written in SMART format), state standards and Department of Diné Education (DODE) standards, materials needed, and activities. Cultural adaptations will be informed by the formative assessment, along with project team members who know and practice traditional teachings and have experience teaching in elementary education, and work with school partners. Adaptations of materials from previous curricula will also address the learning needs and digital media platforms of today’s youth (33).

Curriculum fidelity will be monitored systematically post-lesson using fidelity checklists (34). Teachers will be asked to fill out a checklist utilizing a likert-scale feedback form that covers four primary constructs: adherence, dosage, quality of delivery, and participant responsiveness. Adherence will measure quality and thoroughness to the original lesson. Dosage will cover components such as number of students taught in lesson, frequency of the lesson, and time length of each lesson. Quality of the delivery covers the quality and thoroughness in preparation, skills, leadership, and enthusiasm. Lastly, participant responsiveness will capture student engagement during the lesson. Teachers will also be given the opportunity to provide written feedback regarding unforeseen circumstances such as changes in schedule or group dynamics that might affect lesson delivery (see Table 2).

**Table 2:** Overview of P2 classroom curriculum.

Lesson title	Time	Objectives	Activities
Lesson 1: Introduction to P2: Goal Setting for Nutrition and Physical Activity	35–45 min	(1) Describe two ways how Navajo ancestors lived a healthy life. (2) Demonstrate with their bodies, the importance of energy balance. (3) Identify two sources of energy in and energy out. (4) Identify and write one personal energy balance goal.	(1) Introduction to P2 (2) Discussion–Compare and Contrast Healthy Living Now and Then (3) Hands on Activity–Energy Balance (4) Worksheet–Set an Energy Balance Goal
Lesson 2: Being Physically Active	30–45 min	(1) Be able to describe at least one way that physical activity affects or benefits the body. (2) Name 3 ways to be physically active. (3) Name and describe two body cues they may feel after being physically active.	(1) Video and Discussion of Physical Activity Effects and Benefits (2) Hands on Activity–Movement Bingo and Body Cues (3) Set a Class Goal to Perform Brain Breaks in Class
Lesson 3: Healthy Foods Give You Power	35–45 min	(1) Identify at least two healthy foods from each of the five food groups. (2) Name one way healthy foods help the body grow and have energy. (3) Demonstrate appropriate portion sizes for foods using hand-based measurements (palm for protein, two hands for veggies, etc.).	(1) Introduction and Video–“Food Groups for Kids” (2) Discussion–Navigating ‘My Native Plate’ (3) Portion Sizing Using Your Hand (4) Create Your Own My Native Plate
Lesson 4: Taking Care of Your Feelings	25–30 min	(1) Identify two different emotions and describe what these emotions feel like. (2) Identify two ways to manage stress and calm down their minds and bodies.	(1) Video & Discussion: Understanding and Expressing Emotions (2) Importance of Breathing and Practicing Breathing Techniques (3) Journaling (4) Class Discussion
Lesson 5: Moving Your Body Makes You Strong	40 min	(1) Be able to demonstrate how to check their heart rate (radial side of wrist or carotid side of neck) on their body. (2) Demonstrate two exercises that can make their body strong. (3) Complete a goal setting worksheet stating at least three exercises they can do outside of the classroom.	(1) Storytelling–Daryl & Amanda Start a Physical Activity Plan (2) Hands on–Measuring Heart Rate After Exercise (3) Making a Physical Activity Goal
Lesson 6: Getting Enough Sleep	40–45 min	(1) Explain why sleep is important for their bodies. (2) Identify two healthy bedtime habits. (3) Explain the importance of limiting screen time.	(1) Video & Discussion–Why do we need sleep? (2) Understanding Screen Time (3) Creating Healthy Bedtime Habits by Setting a Goal
Lesson 7: Traditional Foods are Healthy	60 min	(1) Explain why traditional foods are important to culture, family and community. (2) Name two traditional foods and their significance.	(1) Introduction of Taste Testing Event with an Native American Chef (Chef Denella, a Navajo Chef from Tuba City) (2) Taste Testing New Snacks (3) Debrief and Thank-You Card (To be done after event)
Lesson 8: Making Healthy Choices	45–50 min	(1) Identify 2 out of the 4 key components of a nutrition label. (2) Compare and contrast nutrition labels of different foods. (3) Identify the healthiest food choice among snacks and drinks. (4) Explain two reasons why water is a healthier choice than sugary drinks.	(1) Comparing Nutrition Facts on Snacks and Drinks (2) Importance of Water (3) Investigating the Amounts of Sugar
Lesson 9: Building a Healthy Future	30–40 min	(1) Identify each of the 5–2–1-0 concepts. (2) Name at least 2 healthy habits that they can share with their families to help create a healthier future.	(1) Introducing the 5–2–1-0 Code (2) Hand On Activity–5-2-1-0 Get up and Move Review (3) Worksheet 1–Tracking Your 5–2–1-0 Habits
Lesson 10a: P2 Wellness Showcase: What We Learned, What We Live–Part 1	40–45 min	(1) Recall key concepts or topics from the last nine lessons by creating a graphic organizer. (2) Transfer key concepts into creating a poster of drawings and key words learned in the curriculum.	(1) Review P2 Lessons (2) Graphic Organizers (3) Begin Creating Posters
Lesson 10b: P2 Wellness Showcase: What We Learned, What We Live–Part 2	45–60 min	(1) Finish creating a healthy living poster of their own drawings and using language that reflect what they learned from the P2 curriculum. (2) Practice their public speaking skills by sharing 1–2 concepts from their poster that they learned well.	(1) Complete Creating Posters (2) Present Posters to Class and Healthy Champion Award

#### 2.5.3 Diné (Navajo) chef consultation

An Indigenous chef will consult with the intervention schools’ food service program to review current menus, particularly the availability of traditional foods, fresh fruit, and vegetables. The review will identify opportunities to incorporate traditional foods into school menus and explore students’ preferences for healthy options, such as fresh fruit and vegetables, to increase their consumption. Further, the chef will lead taste testing sessions with fourth grade students to explore preferences for traditional healthy food recipes.

#### 2.5.4 Wellness policy assessment

Each school’s wellness policy will be assessed at baseline and post-intervention using the Wellness School Assessment Tool (WellSAT). The baseline assessment will identify gaps – whether federally required and recommended elements are addressed, and whether policy uses vague or specific, strong, enforceable language. Baseline findings will be shared with each school’s principal. Because each school is governed by a local school board, project team members will work with the school wellness committee to develop recommended revisions for submission to the school board for adoption. As part of this work, project team members will train the school wellness committee on using the WellSAT tool so schools can independently assess and strengthen their wellness policies beyond the study period.

### 2.6 Recruitment of schools and participants

Principals will be contacted and queried for their interest in study participation. Schools that previously participated in school-based wellness project work will be prioritized, followed by schools located in the Navajo Nation and within a 150–200 mile radius of the institution. For schools that express interest in participating, an in-person meeting will be arranged to provide study information and allow for further discussion and dialogue with principals. Upon each school principal’s agreement to participate, a school board presentation will be arranged to seek approval.

To recruit focus groups and interview school personnel, we will work through school principals to send email invitations to school personnel to elicit their support and encourage participation. For those agreeing to participate, consent procedures will be completed, followed by scheduling of focus groups and interviews at a time and location convenient for participants. Some schools will have Wellness Councils and/or a number of people involved in wellness and others will not. As a result, schools with more than two personnel will have a focus group and those with one will have an interview. Participants will be asked for their approval to audio record; and a script will be followed. Participants will receive a $25 gift card from a local grocery store, and snacks and water will be provided.

In collaboration with school officials, recruitment of fourth grade students will begin with classroom education sessions and an evening school event—the Healthy School Carnival— hosted by the project team and Tribal and Indian Health Service partners. The classroom presentations will include an overview of the study and a ‘show-and-tell’ of project equipment (e.g., Tanita scale, Veggie Meter^®^, and accelerometer) to build student interest and excitement. Students will take home a flyer inviting their families to attend the carnival, where there will be games, dinner, health screening, and education booths. Interested students and their parents or guardians will be invited to a separate room for an orientation to the study, a demonstration of project equipment (for parents), and parental consent and child assent.

### 2.7 Measures

Student baseline and post-intervention data collection will include an array of measurements including biometric assessments such as the Veggie MeterⓇ and accelerometer, height and weight for BMI. In addition, 24-h dietary recalls will be collected from a randomized subset of students. Self-report surveys will be collected that measure students’ perception of the school environment and their diet along with a survey to measure changes in knowledge. Baseline assessments in all schools will bewithin the first few months of the school year, between August and October. Data collection at each school will be conducted 1 week after the Healthy School Carnival, ensuring the study remains fresh in the minds of students, parents, and school personnel. To facilitate an organized approach to data collection, a station diagram mapping each of the measurements will be designed and shared with the study team and school personnel in advance to ensure adequate space is secured, and students move sequentially and efficiently from one station to the next (Figure 1). Post-intervention data collection for students will occur at the end of the school year, starting in March through May for both intervention and control schools. Beyond individual student measurements, a school-level policy assessment will be conducted using WellSAT at baseline and post-intervention.

**Figure 1:**
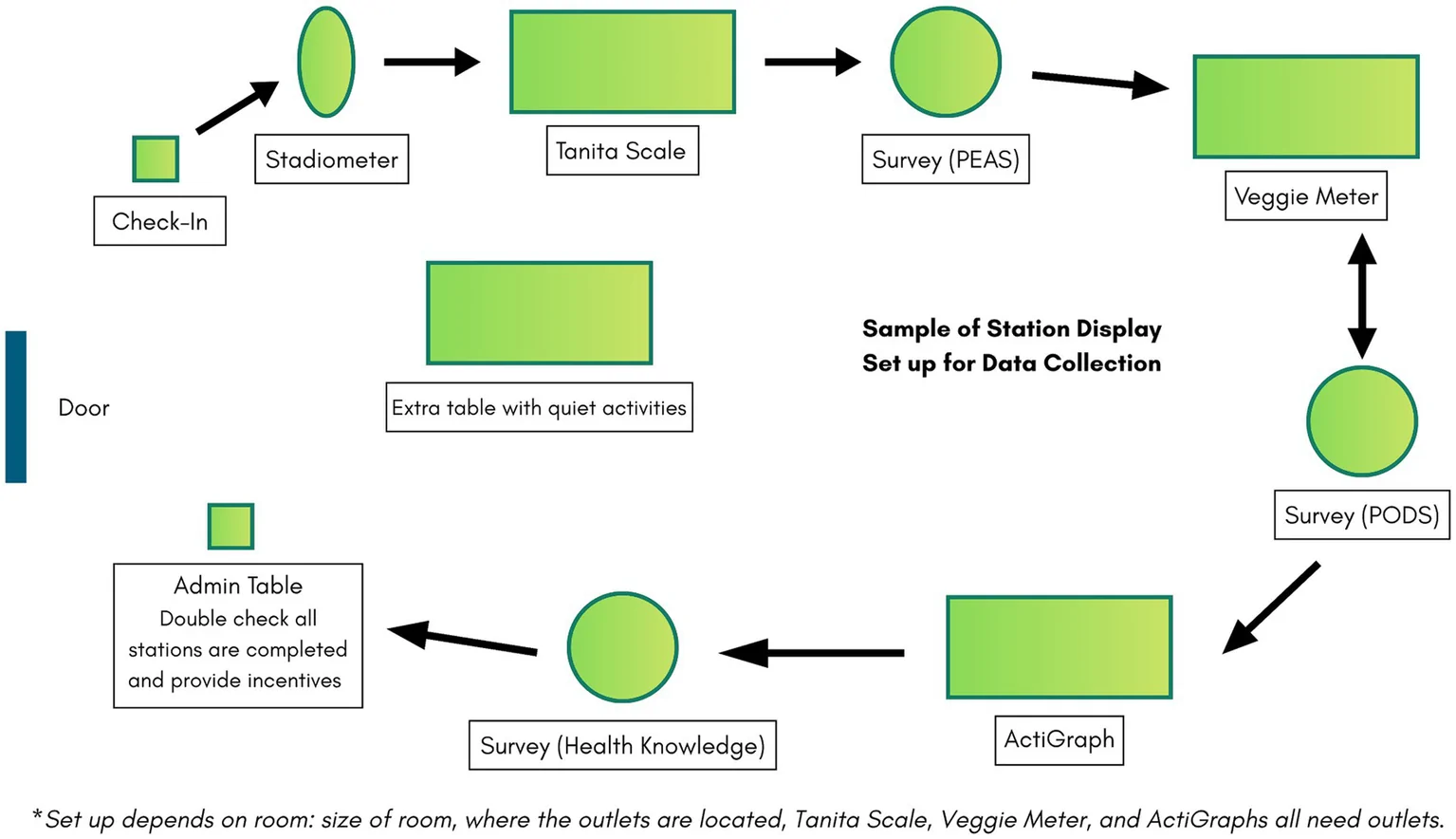
Stations display for data collection.

The primary endpoint is BMI z-score. All secondary endpoints are ordered hierarchically below.

#### 2.7.1 Body mass index

Higher adiposity, or body fatness, is associated with an increased risk for cardiometabolic diseases (1). A high *Body Mass Index (BMI*) can be an indicator of high body fatness (35). BMI is a person’s *weight* in kilograms divided by the square of *height* in meters, and it can be used to screen for weight categories that may lead to health problems (35). Therefore, accurate measurements of height and weight can help assess a person’s risk for health problems and indirectly measure how healthy lifestyle changes can impact one’s health. For youth, BMI measures will be converted to z-scores according to the 2000 CDC Growth Reference (35). Z-scores are adjusted for age and sex and represent standard deviations from the mean (e.g., the 50th percentile equates to a z-score of zero). The cutoff for overweight is the 85th percentile (z-score, +1.036), and the cutoff for obesity is the 95th percentile (z-score, +1.645) (36).

Point in time body weight and height will be measured using a calibrated Tanita SC-331S Body Composition Analyzer^®^ and SECA Stadiometer^®^. The participants should be asked to remove any excess clothing (sweatshirts, sweaters, or jackets) and should be barefoot. The individual should also be asked to remove any items from their pockets. Watches, belts, necklaces, and other jewelry that are of considerable weight should be removed before taking the weight measurement.

Collecting weight and height can produce feelings of self-consciousness. Students may feel self-conscious about taking their shoes off because of odor, condition of their shoes, socks, or feet, or children may be embarrassed about their weight. For these reasons, a designated confidential area portioned from others’ view will be set up. The person conducting height and weight measures should be the same sex/gender as the youth whenever possible.

#### 2.7.2 Fruit and vegetable intake

Carotenoids are fat-soluble pigments found in a wide variety of fruits and vegetables (F/V) that cannot be synthesized by the human body (37, 38). Deep-colored F/V, namely red, orange, green, and yellow, are excellent dietary sources of antioxidants and carotenoids, and serve as established biomarkers of F/V consumption (37). While blood carotenoid concentrations serve as the best measure of F/V intake, the collection process is invasive and labor-intensive, and measures are sensitive to variation in F/V consumption on a day-to-day basis (39). Self-report methods are prone to their own limitations, including unique time burdens and method-specific bias that can result in misestimations of F/V intake (40). Given data collection constraints, techniques incorporating spectronomy have been established and utilized as non-invasive alternatives.

The Veggie Meter^®^ (Longevity Link Corporation, Salt Lake City, UT) was selected for this study for its affordability, portability, and sensitivity to objectively measuring fruit and vegetable consumption via carotenoids. It is a non-invasive device that uses reflection spectroscopy to measure the level of carotenoid pigments in a person’s skin. Scanning the tip of the finger with a white LED produces a score that scales linearly with tissue carotenoid levels. Higher scores reflect higher carotenoid levels and, correspondingly, greater F/V intake (40, 41). Further, changes can be tracked over time in response to changes in diet. Due to carotenoid levels differing slightly between fingers, it is advisable to always measure the same finger when tracking changes (42).

A laptop computer interfaces with the Veggie Meter^®^ to analyze the light reflected from the fingertip, obtaining a carotenoid score using proprietary software. The measurement takes 10 or 45 s, depending on whether a single- or multiple measurement mode is chosen. In multiple measurement mode, the finger is inserted and retracted three times and an average score is determined from the three measurements (42). The present study will use the multiple measurement mode.

#### 2.7.3 Physical activity tracking

Moderate to vigorous physical activity (MVPA) is a category of activity intensity that is measured using metabolic equivalents (METs), defined as the ratio of active to resting metabolic rate (43). Moderate-intensity activity is defined as 3–6 METs, and vigorous-intensity activity is defined as any activity above 6 METs; thus, MVPA overall is any activity exceeding 3 METs (44). The U. S. Department of Health and Human Services recommends that children accumulate at least 60 min of MVPA daily, with individual activity bouts lasting greater than 10 minutes (45). Although meeting these guidelines is ideal, shorter bouts under 10 min may still offer protective factors against certain chronic conditions (46).

Previous studies of school-aged children have used accelerometers to measure MVPA and standing time (47, 48). However, the use of accelerometry in physical activity research within American Indian communities is limited. Teufel-Shone et al. (49) found no use of accelerometers across 64 physical activity interventions between 1986 and 2006, and more recently, Kokenge et al. (50), identified only 6 of 25 intervention studies that employed accelerometers published between 2006 and 2021. Both reviews call for more culturally appropriate and rigorous measurement practices in physical activity research within AIAN communities.

In the present study, physical activity will be measured over a 7-day period using ActiGraph^®^ wrist accelerometer. Accelerometers are small, battery-operated devices that can be placed on the wrist, ankle, or waistline and capture movement data through motion sensors (51). For this study, the devices are worn on the wrist of the non-dominant hand. Acceleration is measured in three planes: forward/backward (anterior–posterior); up/down (vertical); and side-to-side (medial-lateral) (51). Accelerometers provide objective measures of activity that provide “real-time” assessment of intensity, duration, and frequency of activity (51). The ActiGraph^®^ GT3X + records continuous, high resolution acceleration data, which are converted into a variety of objective physical activity and sleep metrics within the Actilife software. The Chandler Children cutpoints, which were derived from a sample of 8–12 year olds who wore a wristworn GT3x + on their non-dominant wrist, will be used for activity intensity classification and will quantify time spent in MVPA (52).

Non-wear time is detected via default Choi algorithm (53) within Actilife. Valid wear time is defined as ≥ 4 days, with ≥10 h of daily wear time, including 1 weekend day (54). Actilife generates variables on a daily level, allowing exclusion of days with <10 h wear time before calculating average MVPA. Available accelerometry data that fails to meet the threshold of validity could be included or excluded in sensitivity analyses.

#### 2.7.4 Dietary recall

The 24-h dietary recalls consist of 3 non-consecutive days (2 weekdays and 1 weekend day) per participant at each measurement time point. For budgetary reasons, a randomized subset of enrolled students will participate in these recalls. Trained interviewers from the Behavioral Measurement and Intervention Shared Resource (BMISR) conduct these approximately 30-min interviews by phone using the USDA multi-pass method and enter the data into a database using the Nutrition Data System for Research software version 2025 (NDS-R), developed by the Nutrition Coordinating Center (NCC), University of Minnesota, Minneapolis, MN. NDS-R is a microcomputer-based system for collection and analysis of dietary data that prompts the user to describe food intake at the level of detail such as food source, processing method, fat and salt used in preparation, and ingredients that contribute to fat and sodium intake (55).

The nutrient database contains over 18,000 foods, 7,500 brand-name products, and many ethnic foods. Values are available for 174 nutrients and nutrient ratios. The database is derived from USDA, food manufacturers, foreign food composition tables, and scientific literature. The NDS-R has the ability for simplified food grouping at the whole food or ingredient level (55).

Interviewers are trained to use probing questions to help children recall eating occasions and what they ate such as asking where they were and who they were with. The interviewers also have access to the school breakfast and lunch menus to cue participants if needed. In addition, a Food Amounts Booklet is provided to each participant for use during their phone interviews. The booklet depicts food and abstract shapes and figures in various sizes to help with portion estimates (56).

#### 2.7.5 Student perceptions of school physical activity and nutrition environment

The Perceptions of Environment at School (PEAS) and Patterns of Diet at School (PODS) comprise a two-part survey for students designed to assess outcomes of school-based obesity interventions. To our knowledge, this is the first time the PEA-PODS survey will be used among American Indian students in Tribal schools. This tool will provide a more comprehensive understanding of how school environments shape student health behaviors (57, 58). PEAS contains 4 subscales related to perceptions of policies/practices of: (1) school physical activity and nutrition, (2) teacher and classroom, (3) cafeteria, and (4) recess. Items in the first and last subscale have Likert-type response options (“never” to “always”). We slightly rephrased questions in the second subscale to refer to “My teacher” rather than “Teachers at my school” because we thought this was better suited to our elementary-school setting, where students have predominantly one main classroom teacher. We also modified response options in subscales 2 and 3 to “no,” “yes,” and “I don’t know” for simplicity.

PODS assesses location, frequency, and type of foods consumed at breakfast and lunch during a typical school week. We administered only a subset of PODS questions to reduce survey fatigue and focus on dietary consumption of school-provided lunch, since additional details regarding broader dietary intake will be measured separately via dietary recall phone calls. PODS questions relate to type, amount, and frequency of eating school lunch items (e.g., main item, fruits, vegetables, beverages).

#### 2.7.6 Health knowledge survey

An age-appropriate health knowledge survey will be developed to directly assess the classroom-based health education intervention. It will draw from two existing validated instruments designed for similar fourth-grader populations: the Pathways study (18, 59) and California’s Power Play Campaign (60). This approach will allow for capturing the full complexity of content, while remaining aligned with the project’s primary outcomes to improve BMI, fruit and vegetable knowledge and intake, and physical activity. It also permits adaptations that can reflect the Diné (Navajo) cultural context of the current study, curriculum content informed by formative assessment findings, and state and the Department of Diné Education cultural values standards. The survey will primarily address knowledge and behavior.

#### 2.7.7 Wellness school assessment tool (WellSAT)

The Wellness School Assessment Tool (WellSAT 3.0) is a widely used, reliable online instrument for evaluating the strength and comprehensiveness of school wellness policies across six categories that correspond with state and national standards. WellSAT is continuously updated to reflect current standards, including federal requirements, nutrition environment, nutrition education, physical activity, employee wellness, and integration and coordination (61, 62).

Each policy item will be scored on a 0–2 scale: 0 indicates the topic is not addressed in policy; 1 indicates the topic is addressed vaguely or not clear; and 2 indicates the topic is clear, specific, and contains strong language. Upon completion, the tool will generate a comprehensive and strength score for each wellness category along with an overall wellness policy score. The comprehensive score will reflect the proportion of policy items that are addressed, while the strength score will reflect the proportion of required policy topics written in clear, specific language (61). Baseline WellSAT results will be shared with schools to guide them in updating their wellness policies, and post-intervention assessments will be completed at the end of the school year.

### 2.8 Analysis

#### 2.8.1 Qualitative analysis

The team will receive training from a principal investigator on qualitative data coding and analysis through presentations, readings, and videos explaining qualitative data, biases, coding, analysis, interpretation, organization, and storage. Data analysis will involve a process of familiarization, coding, identifying themes, revisiting themes, defining and naming themes, and summarizing the findings (63). First, team members will familiarize themselves with the data by thorough reading of transcripts, and as needed, re-listening to the original recordings. Second, inductive line-by-line coding will be conducted by identifying initial codes from participants’ words or phrases. Third, initial codes will be revisited and grouped to generate emerging categories. Fourth, each team member will present their line-by-line codes to the rest of the team and offer suggestions for categories and their rationale for the category. The team will then discuss differing viewpoints and decide whether to keep, divide, discard or create the suggested category, which is a process that leads to the creation of themes. Finally, themes will be reviewed to ensure themes are distinct and clearly defined for written reporting and dissemination to partners.

#### 2.8.2 Statistical methods

Youth measurements will occur at baseline (beginning of academic year: August–October) and end-of-study (end of academic year: April–May) for both arms. Each outcome measure (e.g., BMI z-score, minutes of MVPA, and carotenoid intake score) will be summarized, and change from baseline will be calculated. The effect of the intervention will be tested using linear random-effects models to adjust for the baseline values and allow for correlation among the measurements of students within a school. Transformations will be explored, as needed, to meet the normality assumptions of the random effects models. All analyses will report and interpret the estimated coefficients with 95% confidence intervals. A sensitivity analysis will be performed using random-effects models with small sample adjustment. Missing data will not be imputed. Students who are lost to follow-up will still have their baseline measurements included in the mixed-effects models. The primary endpoint (BMI z-score) will be tested using an alpha level of 0.05; the main secondary endpoints (carotenoids, MVPA) will be tested using a Bonferroni-adjusted alpha level of 0.025 (0.05/2). Data will be analyzed using Stata 18 (StataCorp, College Station, TX).

## 3 Discussion

Despite many decades of childhood obesity prevention initiatives and research, alarmingly, no studies to date have yet made a meaningful difference in improving BMI among American Indian youth (45). The health risks associated with excess weight are deeply concerning for present and future generations, particularly the links between obesity, Type 2 diabetes. and cancer, and the emerging reality that cancer is now the leading cause of death in individuals with Type 2 diabetes (64). This underscores the critical importance of the current study, which prioritizes primary prevention of obesity with school-age children in rural Tribal primary schools, where early intervention holds far greater promise than treatment at later stages in life (65).

Although school-based obesity prevention research has been conducted in Tribal communities, none have leveraged the current federal policy context, specifically the Healthy Hunger-Free Kids Act of 2010 and its mandates to support healthy school environments, making the current study innovative in the school-based research landscape. More specifically, several features of the intervention design warrant discussion.

First, the multi-component structure makes certain that nutrition and physical activity are addressed across multiple levels of the school environment – the classroom, the cafeteria and policy– rather than in isolation. Second, integrating an Indigenous chef into the school food services component grounds the intervention in cultural meaning by connecting cafeteria experiences to culture and to the nutrition content taught in the classroom. Third, the inclusion of a school wellness policy (SWP) assessment moves beyond school health programmatic change to influencing institutional change. An SWP is a federally required structure that is already in place, providing an incredible opportunity to embed and strengthen healthy eating and physical activity into school-wide practices that can be sustained beyond the study. This optimizes the benefits to the respective schools for time and effort invested. Together, these components will work to create a comprehensive, supportive school health environment.

Another unique aspect of this study is the comprehensive assessment approach that will employ robust measures in a school setting to evaluate nutrition and physical activity knowledge and behaviors, diet and fruit and vegetable consumption, physical activity movement and intensity, and perceptions of school environment among fourth grade students. Notably, to our knowledge, this study represents the first-time use of PEAS and PODS surveys among American Indian students in Tribal schools, which are tools that will capture how school environments influence student behaviors (59, 60). Classroom practices and teacher behaviors have been shown to be significant in shaping student health behaviors (66). These data will allow us to assess intervention impact by examining how changes in the school environment relate to student dietary behaviors (59, 67).

Similarly, the use of the WellSAT instrument represents the first application with schools in Tribal communities. WellSAT can provide valuable insights into how school wellness policies measure against recommended standards (61), while also providing a mechanism to determine whether written policies are actually being implemented. Perhaps, most importantly, is the opportunity for schools to develop unique, culturally grounded policies, such as those that can emerge from the Indigenous chef’s collaboration with the school food services program staff.

The rural, remote Tribal setting of this study presents several important limitations that need to be acknowledged. First, rural schools are generally smaller with low student enrollment, which leads to smaller sample sizes and precludes the generalizability of findings beyond the target population being studied (68, 69). Second, rural schools often struggle with limited resources and capacity, such as low teacher recruitment, high turnover, and/or high workloads, and all which can influence successful implementation (70).

In sum, this study protocol outlines a multi-component health promotion curriculum that will be designed and implemented with school partners aimed to improve nutrition and physical activity knowledge and behaviors among fourth grade students. Importantly, it highlights how leveraging school wellness policy can be a promising approach to improving health knowledge and behaviors among American Indian students. Led by Indigenous perspectives, especially the Diné concept of k’é, the project promotes circular relationships among research staff, teachers, school staff and administration, and community partners, ensuring awareness of local priorities and worldviews.
